# Anthropogenic Debris Ingestion by Avifauna in Eastern Australia

**DOI:** 10.1371/journal.pone.0158343

**Published:** 2016-08-30

**Authors:** Lauren Roman, Qamar A. Schuyler, Britta Denise Hardesty, Kathy A. Townsend

**Affiliations:** 1 Institute for Marine and Antarctic Studies, University of Tasmania, Hobart, Tasmania, Australia; 2 School of Biological Sciences, The University of Queensland, St. Lucia, Queensland, Australia; 3 Oceans and Atmosphere Flagship, CSIRO, Hobart, Tasmania, Australia; 4 Moreton Bay Research Station, The University of Queensland, Dunwich, Queensland, Australia; Auburn University, UNITED STATES

## Abstract

Anthropogenic debris in the world’s oceans and coastal environments is a pervasive global issue that has both direct and indirect impacts on avifauna. The number of bird species affected, the feeding ecologies associated with an increased risk of debris ingestion, and selectivity of ingested debris have yet to be investigated in most of Australia’s coastal and marine birds. With this study we aim to address the paucity of data regarding marine debris ingestion in Australian coastal and marine bird species. We investigated which Australian bird groups ingest marine debris, and whether debris-ingesting groups exhibit selectivity associated with their taxonomy, habitat or foraging methods. Here we present the largest multispecies study of anthropogenic debris ingestion in Australasian avifauna to date. We necropsied and investigated the gastrointestinal contents of 378 birds across 61 species, collected dead across eastern Australia. These species represented nine taxonomic orders, five habitat groups and six feeding strategies. Among investigated species, thirty percent had ingested debris, though ingestion did not occur uniformly within the orders of birds surveyed. Debris ingestion was found to occur in orders Procellariiformes, Suliformes, Charadriiformes and Pelecaniformes, across all surveyed habitats, and among birds that foraged by surface feeding, pursuit diving and search-by-sight. Procellariiformes, birds in pelagic habitats, and surface feeding marine birds ingested debris with the greatest frequency. Among birds which were found to ingest marine debris, we investigated debris selectivity and found that marine birds were selective with respect to both type and colour of debris. Selectivity for type and colour of debris significantly correlated with taxonomic order, habitat and foraging strategy. This study highlights the significant impact of feeding ecology on debris ingestion among Australia’s avifauna.

## Introduction

Contamination of marine and coastal environments by plastics and other non-biodegradable anthropogenic debris has become an increasing problem facing marine environments worldwide [1–3], and is known to affect to hundreds of species [4]. The recorded effects of anthropogenic debris consumption on fauna include physical damage to the digestive tract [5], reduced food consumption due to lower available stomach volume and therefore poorer fat deposition and body condition [6,7], and obstruction of the digestive tract which may result in starvation [8]. Additional risks of anthropogenic debris ingestion include the transfer of pollutants [9] and bioaccumulation of plastic-derived chemicals in body tissues [10], toxicity via uptake of persistent organic pollutants (POPs) absorbed by plastic particles [2,11,12], and the translocation of microscopic plastics to other organ systems [13].

Marine birds are widely known to ingest debris: 164 seabird species have been recorded to ingest marine debris globally [4], making marine avifauna a high risk group for both lethal and sub-lethal effects of ingestion. Despite the high frequency of anthropogenic debris ingestion reported in seabirds, debris ingestion is poorly studied in birds that live and forage in coastal and freshwater environments. There are currently limited diet studies that have investigated debris ingestion in shorebird species [7,14]. This paucity of research leaves a large gap in the understanding of whether coastal species ingest anthropogenic debris encountered in their environments.

In Australia, the extent of anthropogenic debris ingestion among marine and coastal birds is poorly known. Though there have been a numerous records of debris interaction in Australasian avifauna [15], only six of the over 200 known marine and coastal bird species have been systematically investigated for debris ingestion [5,16–20]. Of investigated species, several showed a high frequency of debris ingestion among individuals; Short-tailed shearwater (*Ardenna tenuirostris*), 67–100% [5,19], Flesh-footed shearwater (*Ardenna carneipes*), 90% [17], Wedge-tailed shearwater (*Ardenna pacificus*), 21%, Common diving petrel (*Pelecanoides urinatrix*), 11.7% [20], Kelp gull (*Larus dominicus*) 54% and Pacific gull (*Larus Pacificus*), 2% [16]. This ubiquity of marine debris ingestion among the few species investigated highlights the need to understand the prevalence of anthropogenic debris ingestion among the remaining Australian avifauna.

To address the gap in knowledge regarding marine debris ingestion among most Australian marine-associated bird species, we conducted a broad survey of marine, aquatic and coastal bird species in eastern Australia. We sampled species from nine taxonomic orders, five habitat groups and six feeding strategies. Ecological factors such as diet and foraging behavior [21] have been associated with increased occurrence of debris ingestion; and certain debris items (e.g. hard plastics) and colours (e.g. whites and light colours) are commonly encountered in ingested samples [19,22,23]. It is largely unknown if these debris items are more prevalent in gut contents due to selectivity by fauna, or due to an abundance in the environment. Several recent studies investigated selectivity of ingested anthropogenic debris by comparing items ingested to background availability. These studies demonstrated selectivity in debris ingestion by Short-tailed shearwaters [19], Hawksbill sea turtles (*Eretmochelys imbricata*) and Green sea turtles (*Chelonia mydas*) [24].

We aim to address the question of whether ingested debris items are subject to selectivity by exploring whether marine birds select debris with particular physical characteristics relative to what is available in the marine environment, and whether foraging ecology correlates with debris ingestion likelihood. Through extensive surface trawling of anthropogenic debris in a range of locations throughout the sampled species foraging range, we provide a spatial and temporal snapshot of the debris available to foraging marine birds.

## Methods

**Fig 1:**
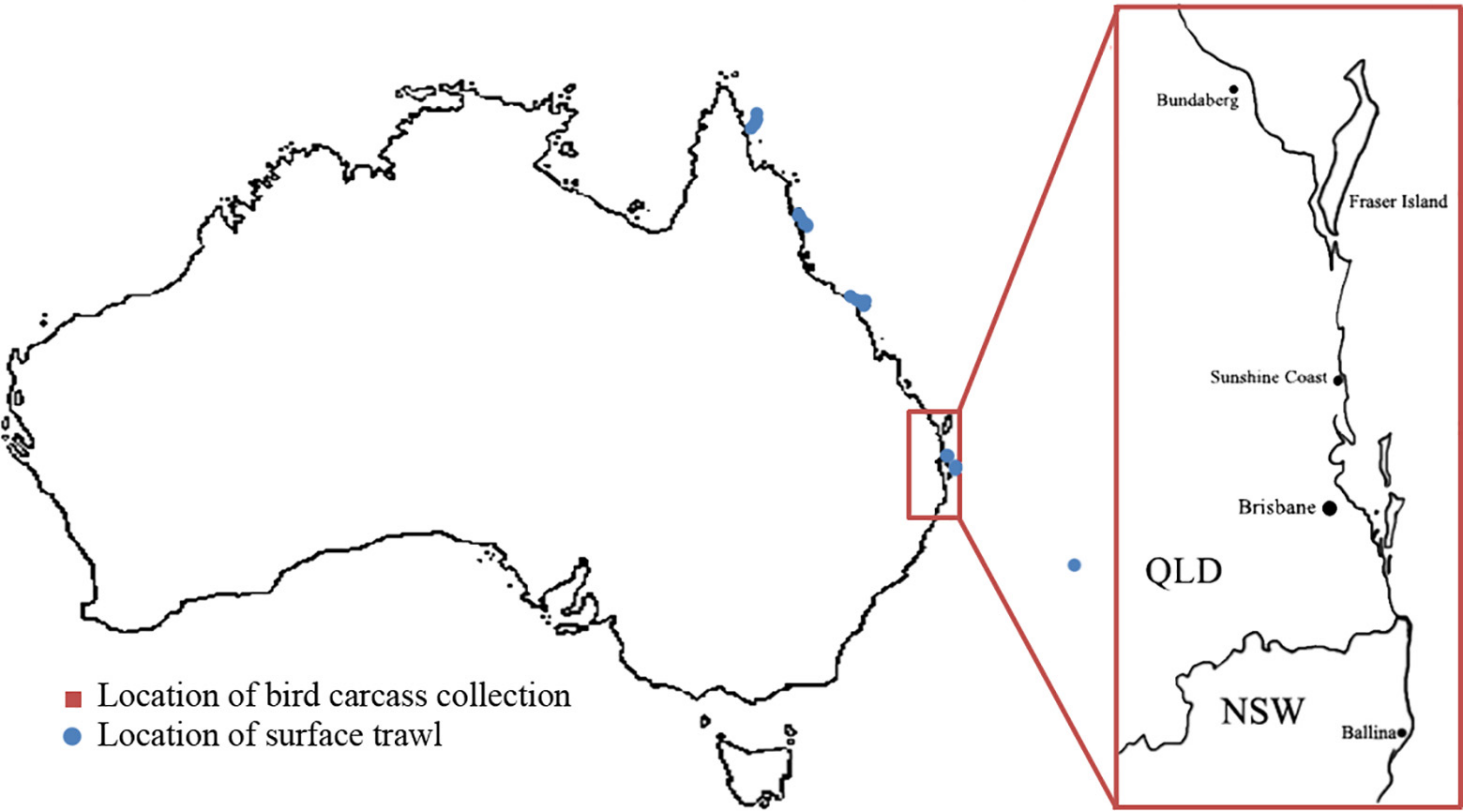
Map of eastern Australia showing approximate of where birds used in the study were collected. Fraser Island, QLD (24°42' S, 153°15' E) at the northern end of the study range, and Ballina, NSW (28°51' S, 153°33' E) at the southern end. Blue circles represent the locations where surface trawls were undertaken.

Marine and coastal birds were obtained from a variety of sources in eastern Australia located between Fraser Island, Queensland (24°42' S, 153°15' E) and Ballina, New South Wales (28°51' S, 153°33' E) (Fig 1) within 10 km of a marine environment between March and October 2013 (Scientific purposes permit no.WISP12620313, Department of Environment and Heritage Protection, Queensland Government). Birds were opportunistically collected dead by permitted people, or injured birds collected and transferred to wildlife hospitals by the public were collected by the authors if birds died in care. Freshly deceased birds were immediately frozen at -20°C or refrigerated at 5°C if the necropsy was to be performed in less than 48 hours.

Anthropogenic debris in the marine environment available to foraging marine birds was determined by sampling a snapshot of debris in a region in eastern Australia in which the collected birds would likely be foraging. A total of 90 surface trawls for marine debris were conducted between far north Queensland (-12° 2'28 S, 143°58'5 E) and Lord Howe Island, New South Wales (-31°31'42 S 159°3'39 E), between November 2011 and May 2012. Surface trawls of between 12 to 30 minutes were conducted on a variety of small craft travelling between 3–6 knots, using a manta trawl net with a mouth size of 600x200 mm and a mesh size of 300μm. Samples were soaked in sea water for a minimum of 30 minutes to retrieve any floating plastics, then sieved through a series of sieves from 1 cm to 330 μm to identify small particulate marine debris. Debris type, colour, size, and buoyancy, were recorded for each item (Schuyler et al. unpublished data).

Necropsy has been shown to be the most effective and comprehensive method of surveying ingested plastic [21]. Therefore, to investigate anthropogenic debris ingestion in eastern Australian marine, aquatic and coastal birds, we carried out entire gastro-intestinal content necropsies on animals that had either been found dead or had been euthanized due to ill health or traumatic injury.

Necropsy methods followed van Franeker’s collection and dissection procedures manual for northern fulmars [25], whereby the entire proventriculus and gizzard are removed from the bird. Following removal of proventriculus and gizzard, contents were thoroughly inspected. Debris items found in the gastro-intestinal tract were removed with tweezers, washed gently in distilled water, allowed to dry and stored in aluminium foil for later confirmation of material type and sorting.

Anthropogenic debris from birds and from trawl samples was visually sorted into 5 types and 10 colours for analysis. The five type categories were hard plastic (any rigid plastics), soft plastic (any easily malleable plastics, including plastic bags, readily flexible plastics, etc.), balloons, fishing debris (including hooks, fishing line, fishing lures and sinkers) and other (any item that does not fit previous categories, e.g. polystyrene foam and rubber). Colour categories were black, blue, brown, clear/translucent, green, grey, orange, red/pink, white and yellow. Where debris was fouled or stained, the original colour was determined by gently scraping away a small section of the surface with a scalpel blade. Buoyancy was determined by placing debris in seawater, and recorded as positively, neutrally or negatively buoyant.

Birds were grouped for analysis according to taxonomic order, habitat and feeding behavior. Species identification followed Bird Life International’s taxonomic checklist (Version 7) [26]. Five habitat groups were allocated based upon the species’ habitat and primary foraging location as identified by Croxall et al., Higgins and Davies and Marchant and Higgins [27–31]. Habitat types were defined as: “pelagic” (birds that forage in open ocean), “coastal marine” (birds that feed over or in near-shore marine environments), “coastal” (birds that forage on land along the shoreline, mangroves, or similar land or intertidal coastal environments), “fresh water” (birds that predominantly forage in fresh water bodies and wetlands), and “open grasslands” (coastal birds that forage open grassy/coastal habitat) [27–31].

Six primary feeding behaviour groups were allocated based upon feeding behaviours identified by Croxall et al., Higgins and Davies, and Marchant and Higgins [27–31]. Feeding behaviours were defined as: “surface feeding” (predominantly feeding at or near surface of ocean, employing foraging strategies such as flight feeding, surface feeding and surface diving species), “plunge diving” (feeds predominantly by diving from air followed by near-surface underwater pursuit of prey), “pursuit diving” (underwater pursuit of prey, initiated underwater, at all depths), “bill searching” (sensory, non-visual foraging by sensing prey with bill), “grazing” (feeding on vegetation/algae) and “search-by-sight” (visual searching and pursuing prey on foot in terrestrial environments) [27–31].

To determine whether marine birds (defined as species inhabiting pelagic, coastal marine and coastal habitats) exhibit selectivity, we compared the types and colours of debris ingested by birds to debris obtained in surface trawls, as per Acampora et al. 2014 [19]. While this sampling method does not perfectly represent the individual items each bird will interact with along its foraging path, to date, it is the best available methodology for comparing items ingested at sea with those available in the environment. To determine whether taxonomic order, habitat and foraging ecology correlated with the type and colour of debris items selected, we assessed whether the types and colours of ingested debris differed significantly within taxonomic orders, habitat groups, and foraging behaviour groups.

### Statistical Analysis

All statistical analyses were performed using statistical software R (R version i386 3.0.2) [32]. To determine whether debris ingested by seabirds of differing life history categories differed significantly from background availability, we conducted a permutational multivariate analysis of variance using the community ecology R package ‘vegan’ [33]. We determined the Bray-Curtis dissimilarity between ingested debris and available background debris, then applied a linear model to the dissimilarity value matrices, using a permutation test with pseudo F-ratios. Categorical groupings represented by less than 3 samples or where ecological data were unknown were excluded from analysis. Permutational multivariate analysis of variance analysis was also used to detect significant variation in the types and the colours of anthropogenic debris within the taxonomic order, habitat and feeding method groups.

## Results

### Prevalence of anthropogenic debris ingestion among marine avifauna

In total, 378 individual birds were sampled, representing 61 species across nine taxonomic orders. Of those, 89 individual birds (23.5% of total) ingested 479 items of debris, with individuals ingesting between 1–26 items (mean = 5.8 items, median = 3 items, SE ± = 0.67).

Of the species investigated, 19 (30.6%) had consumed anthropogenic debris (Table 1). Order Procellariiformes was the most likely to contain species that consumed debris, with 65% of species surveyed having ingested debris, and 44.1% of necropsied individuals. Orders Suliformes, Charadriiformes and Pelecaniformes also contained species that ingested debris. There was no anthropogenic debris recorded in the Accipitriformes, Anseriformes, Gruiformes, Phaethontiformes or Podicipediformes sampled (Table 1).

**Table 1:** Taxonomic distribution of marine debris ingestion in necropsied seabirds.

Order	Family	Species	*Scientific name*	Habitat	Foraging	*n* individuals with ingested debris (*n* necropsied)	Mean ingested debris items per individual	Sum of ingested debris items				
								Hard Plastic	Soft Plastic	Balloon	Fishing	Other
**Accipitriformes**						**0 (7)**						
	***Accipitridae***	Brahminy Kite	*Haliastur indus*	Coastal Marine	Surface feeding	(2)						
	***Accipitridae***	White Bellied Sea Eagle	*Haliaeetus leucogaster*	Coastal Marine	Surface feeding	(2)						
	***Pandionidae***	Osprey	*Pandion haliaetus*	Coastal Marine	Surface feeding	(3)						
**Anseriformes**						**0 (31)**						
	***Anatidae***	Black Swan	*Cygnus atratus*	Fresh water	Grazing	(8)						
	***Anatidae***	Hard Head	*Aythya australis*	Fresh water	Grazing	(1)						
	***Anatidae***	Pacific Black Duck	*Anas superciliosa*	Fresh water	Grazing	(5)						
	***Anatidae***	Plumed Whistling Duck	*Dendrocygna eytoni*	Fresh water	Grazing	(5)						
	***Anatidae***	Wood Duck	*Chenonetta jubata*	Fresh water	Grazing	(12)						
**Charadriiformes**						**3 (62)**						
	***Burhinidae***	Bush Stone Curlew	*Burhinus grallarius*	Open grassland	Search-by-sight	**1 (3)	0.66	1				1
	***Charadriidae***	Masked Lapwing	*Vanellus miles*	Open grassland	Search-by-sight	(5)						
	***Charadriidae***	Pacific Golden Plover	*Pluvialis fulva*	Coastal	Search-by-sight	(1)						
	***Haematopodidae***	Pied Oystercatcher	*Haematopus longirostris*	Coastal	Search-by-sight	**1 (1)	20	20				
	***Laridae***	Silver Gull	*Chroicocephalus novaehollandiae*	Coastal Marine	Search-by-sight	1 (12)						
	***Scolopacidae***	Bar Tailed Godwit	*Limosa lapponica*	Coastal	Bill searching	(1)						
	***Scolopacidae***	Sanderling	*Calidris alba*	Coastal	Search-by-sight	(1)						
	***Stercorariidae***	Brown Skua	*Stercorarius antarcticus*	Pelagic	Surface feeding	(1)						
	***Sternidae***	Common Noddy	*Anous stolidus*	Pelagic	Surface feeding	(1)						
	***Sternidae***	Crested Tern	*Thalasseus bergii*	Coastal Marine	Surface feeding	(3)						
	***Sternidae***	Fairy Tern	*Sterna nereis*	Coastal Marine	Surface feeding	(1)						
	***Sternidae***	Little Tern	*Sternula albifrons*	Coastal Marine	Surface feeding	(3)						
	***Sternidae***	Sooty Tern	*Sterna fuscata*	Pelagic	Surface feeding	(27)						
	***Sternidae***	Unknown Juv tern	*Sterna sp*.		Surface feeding	(2)						
**Gruiformes**						**0 (10)**						
	***Rallidae***	Dusky Moorhen	*Gallinula tenebrosa*	Fresh water	Grazing	(2)						
	***Rallidae***	Eurasian Coot	*Fulica atra*	Fresh water	Grazing	(4)						
	***Rallidae***	Purple Swamp Hen	*Porphyrio porphyrio*	Fresh water	Grazing	(4)						
**Pelecaniformes**						**4 (35)**						
	***Ardeidae***	Great Egret	*Ardea alba*	Fresh water	Search-by-sight	(2)						
	***Ardeidae***	Intermediate Egret	*Ardea intermedia*	Fresh water	Search-by-sight	(1)						
	***Ardeidae***	Striated Heron	*Butorides striata*	Coastal	Search-by-sight	(1)						
	***Ardeidae***	White Faced Heron	*Egretta novaehollandiae*	Fresh water	Search-by-sight	(9)						
	***Pelecanidae***	Australian Pelican	*Pelecanus conspicillatus*	Coastal Marine	Surface feeding	4 (15)	0.53		1		6	1
	***Threskiornithidae***	Australian White Ibis	*Threskiornis Molucca*	Coastal	Bill searching	(5)						
	***Threskiornithidae***	Royal Spoonbill	*Platalea regia*	Coastal	Bill searching	(2)						
**Phaethontiformes**						**0 (3)**						
	***Phaethontidae***	Red Tailed Tropicbird	*Phaethon rubricauda*	Pelagic	Plunge Diving	(3)						
**Podicipediformes**						**0 (5)**						
	***Podicipedidae***	Australasian Grebe	*Tachybaptus novaehollandiae*	Fresh water	Pursuit Diving	(5)						
**Procellariiformes**						**76 (169)**						
	***Diomedeiadae***	Buller’s Albatross	*Thalassarche bulleri*	Pelagic	Surface feeding	*2(2)	1		2			
	***Diomedeiadae***	Light Mantled Albatross	*Phoebetria palpebrata*	Pelagic	Surface feeding	(1)						
	***Diomedeiadae***	Shy Albatross	*Thalassarche cauta*	Pelagic	Surface feeding	1 (3)	0.33		1			
	***Diomedeiadae***	Wandering Albatross	*Diomedea exulans*	Pelagic	Surface feeding	(1)						
	***Diomedeiadae***	White Capped Albatross	*Thalassarche steadi*	Pelagic	Surface feeding	(1)						
	***Pelecanoididae***	Common Diving Petrel	*Pelecanoides urinatrix*	Pelagic	Surface feeding	(1)						
	***Procellariidae***	Antarctic Prion	*Pachyptila desolata*	Pelagic	Surface feeding	4 (5)	5.2	25	1			
	***Procellariidae***	Fairy Prion	*Pachyptila turtur*	Pelagic	Surface feeding	*20 (68)	0.49	27	4		1	1
	***Procellariidae***	Fluttering Shearwater	*Puffinus gavia*	Pelagic	Surface feeding	**4 (11)	0.55	4				2
	***Procellariidae***	Gould's Petrel	*Pterodroma leucoptera*	Pelagic	Surface feeding	*2 (2)	1.5	3				
	***Procellariidae***	Little Shearwater	*Puffinus assimilis*	Pelagic	Surface feeding	*2 (2)	2	3	1			1
	***Procellariidae***	Providence petrel	*Pterodroma solandri*	Pelagic	Surface feeding	(1)						
	***Procellariidae***	Salvin’s Prion	*Pachyptila salvini*	Pelagic	Surface feeding	*3 (4)	4	15	1			
	***Procellariidae***	Short Tailed Shearwater	*Ardenna tenuirostris*	Pelagic	Surface feeding	28 (31)	7.65	171	23	31	1	11
	***Procellariidae***	Slender Billed Prion	*Pachyptila belcheri*	Pelagic	Surface feeding	*2 (2)	5.5	8		2		1
	***Procellariidae***	Southern Giant Petrel	*Macronectes giganteus*	Pelagic	Surface feeding	3 (3)	13	37		2		
	***Procellariidae***	Tahiti Petrel	*Pseudobulweria rostrate*	Pelagic	Surface feeding	(1)						
	***Procellariidae***	Wedge Tailed Shearwater	*Ardenna pacificus*	Pelagic	Surface feeding	4 (28)	0.46	12		1		
	***Procellariidae***	Westland Black Petrel	*Procellaria westlandica*	Pelagic	Surface feeding	**1 (1)	2			1		1
	***Procellariidae***	White Necked Petrel	*Pterodroma cervicalis*	Pelagic	Surface feeding	(1)						
**Suliformes**						**6 (56)**						
	***Anhingidae***	Australasian Darter	*Anhinga novaehollandiae*	Fresh water	Pursuit Diving	(8)						
	***Fregatidae***	Lesser Frigatebird	*Fregata ariel*	Pelagic	Surface feeding	(1)						
	***Phalacrocoracidae***	Great Cormorant	*Phalacrocorax carbo*	Fresh water	Pursuit Diving	(2)						
	***Phalacrocoracidae***	Little Black Cormorant	*Phalacrocorax sulcirostris*	Fresh water	Pursuit Diving	1 (7)	0.14					1
	***Phalacrocoracidae***	Little Pied Cormorant	*Microcarbo melanoleucos*	Fresh water	Pursuit Diving	(1)						
	***Phalacrocoracidae***	Pied Cormorant	*Phalacrocorax varius*	Coastal Marine	Pursuit Diving	5 (22)	0.27				6	
	***Sulidae***	Australasian Gannet	*Morus serrator*	Pelagic	Plunge Diving	(13)						
	***Sulidae***	Red Footed Booby	*Sula sula*	Pelagic	Plunge Diving	(2)						
		**Grand Total**				**89 (378)**						

*First debris ingestion record in Australia

**First debris ingestion record in Australia and globally

### Selectivity of debris ingestion

In the 90 oceanic trawls we collected 396 pieces of debris from 52 tows containing debris (range 1–42, mean = 7.6, SE ± = 1.1). The most common items encountered were soft plastics (41 ± 4.9%), followed by “other” debris (28.3 ± 4.8%). Balloons were the least common item found in trawl samples (0.6 ± 0.6%). White (30.7 ± 4.7%) and blue (28.4 ± 2.7%) were the most commonly encountered debris colours in trawl samples.

**Fig 2:**
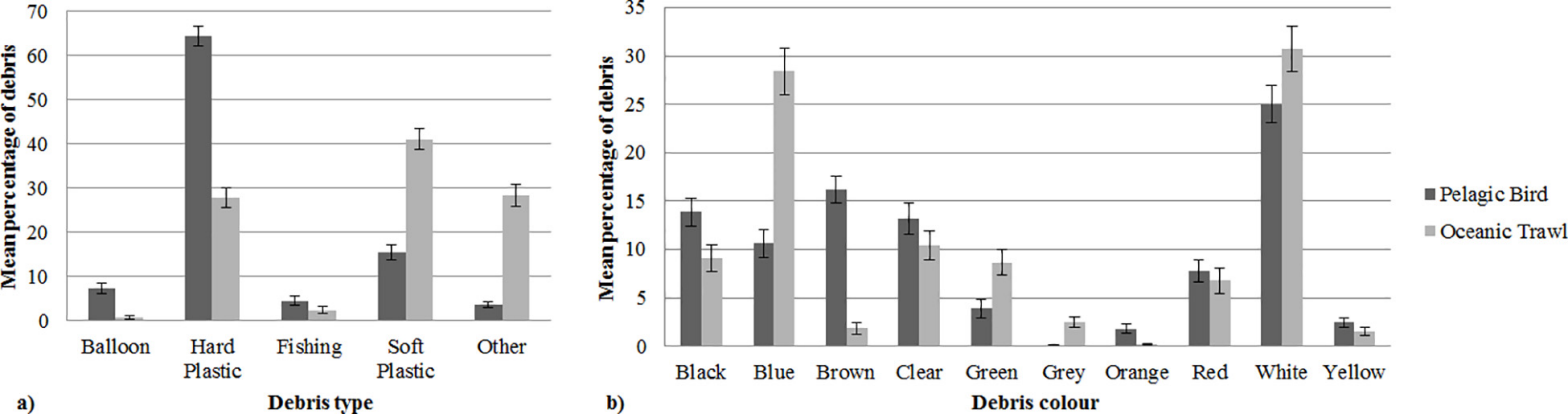
The mean percentage of the (a) type of debris (+SE) and (b) colour of marine debris (+SE) ingested by all marine birds and from all surface trawl samples. The percentage of the type (P = 0.001) and colour (P = 0.001) of marine debris ingested by all marine birds differed significantly from the type and colours of marine debris collected in surface trawls of the ocean off eastern Australia.

The percentage of the types and colours of marine debris ingested by pelagic marine birds differed significantly from the type and colours of marine debris collected in surface trawls in the ocean off eastern Australia (type F-value = 13.912, R^2^ = 0.095, p = 0.001 and colour F-value = 5.417, R^2^ = 0.039, p = 0.001) (Fig 2). Hard plastic debris was the most commonly encountered debris found in marine bird digestive contents (64.26 ± 4.54% of all items ingested), and white was the most frequently ingested colour of debris (24.98 ± 3.83%).

Most debris ingested by birds in this study (95.6%) was positively buoyant, with the exception of fishing line (n = 5), metal fishing hooks (n = 7) and sinkers (n = 2) and ‘other’ debris including a fragment of tile (n = 1), glass (n = 2), and metal wire (n = 2), which were negatively buoyant.

### Selectivity within taxonomic groups

**Fig 3:**
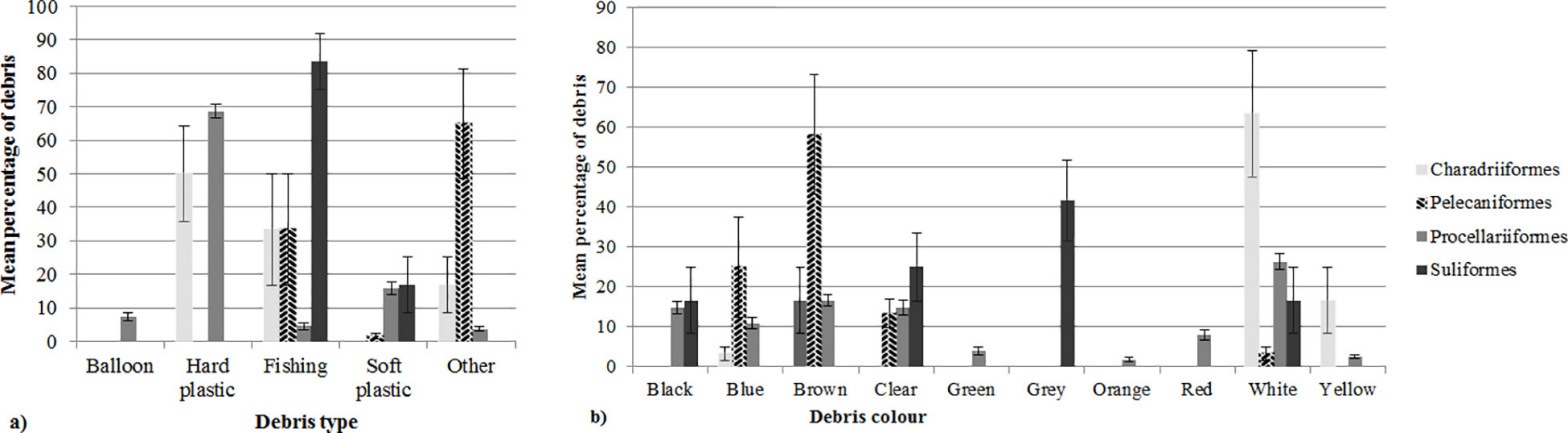
The mean percentage of the (a) type of debris (+SE) and (b) colour of marine debris (+SE) ingested by taxonomic orders of marine and coastal birds. The average combination of the types ingested debris differs significantly (P = 0.001) among taxonomic orders, and average combination of colours of debris differed significantly (P = 0.009) among taxonomic orders.

The combination of both the type and colour of debris ingested differed significantly among taxonomic groups that ingested debris (type F-value = 5.48, R^2^ = 0.17, p = 0.001; colour F-value = 1.86, R^2^ = 0.07, p = 0.009): Procellariiformes (n = 76 /169 individuals, n = 13/20 species), Suliformes (n = 6/56 individuals, n = 2/8 species), Charadriiformes (n = 3/62 individuals, n = 3/14 species) and Pelecaniformes (n = 4/35 individuals, n = 1/9 species) (Fig 3). Hard plastic constituted the greatest percentage of ingested debris in Procellariiformes (68.67 ± 4.36%). The order Procellariiformes were also the only group found to ingest balloons. Hard plastic was also found in Charadriiformes (50 ± 28.87%), but was absent from the digestive contents of Suliformes and Pelecaniformes.

Fishing debris was found in the digestive contents of all seabird orders, but constituted the most abundant ingested debris type only in Suliformes (83.3 ± 16.7%). Soft plastic was ingested by all orders except Charadriiformes. Other debris types, such as cardboard, cloth, wire, glass and rope, were most abundant in Pelecaniformes (65 ± 32.5%), ingested also by Procellariiformes (3.6 ± 0.6%) and Charadriiformes (16.7 ± 8.3%), but not ingested by Suliformes.

### Selectivity within habitat groups

Debris ingestion was found among birds in all habitat types. Species that forage in pelagic habitats had the highest rate of debris ingestion (n = 76/217 individuals, n = 13/27 species), followed by species occurring in coastal marine habitats (n = 10/63 individuals, n = 3/9 species). Debris ingestion was found also in coastal (n = 1/12 individuals, n = 1/7 species), open grassland (n = 1/8 individuals, n = 1/2 species) and fresh water habitats (n = 1/76 individuals, n = 1/16 species). Coastal, open grassland and fresh water habitat groups were not analyzed for selectivity within habitat groupings due to low sample sizes.

**Fig 4:**
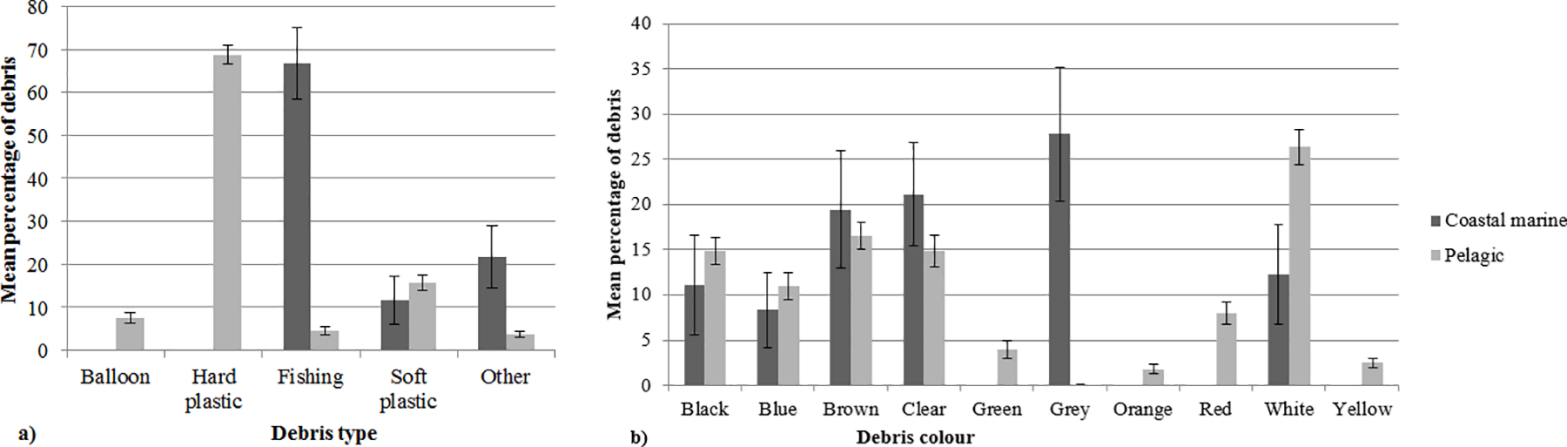
The mean percentage of the (a) type of debris (+SE) and (b) colour of marine debris (+SE) ingested by marine and coastal birds that live in coastal marine habitats (blue bars) and pelagic habitats (red bars). The mean percentage of types of marine debris ingested (P = 0.001) and colour of marine debris ingested differs significantly (P = 0.014) between pelagic and coastal marine habitat types.

The types of marine debris ingested by pelagic seabirds differed significantly (F-value = 11.75, R^2^ = 0.13, p = 0.001) from the type of debris ingested by coastal marine birds. Pelagic birds ingested predominantly hard plastics (68.7 ± 4.4%) while fishing debris (66.7 ± 16.7%) was the most abundant item ingested by coastal marine birds. The colour of marine debris ingested also differed significantly between pelagic and coastal marine birds (F-value = 2.48, R^2^ = 0.03, p = 0.014) (4), though some overlap occurred between colours ingested. Pelagic birds ingested more white, orange, green, red and yellow debris. In contrast, coastal birds ingested more grey items (Fig 4).

### Selectivity within foraging method groups

Debris ingestion was most prevalent in species that forage by surface feeding (n = 80/230 individuals, n = 14/32 species), and was also recorded in species that forage by pursuit diving (n = 6/45 individuals, n = 2/6 species) and search by sight (n = 3/36 individuals, n = 3/10 species). Debris ingestion was not recorded in species that feed by bill searching (8 individuals, 3 species), grazing (45 individuals, 6 species), or plunge diving (18 individuals, 3 species).

**Fig 5:**
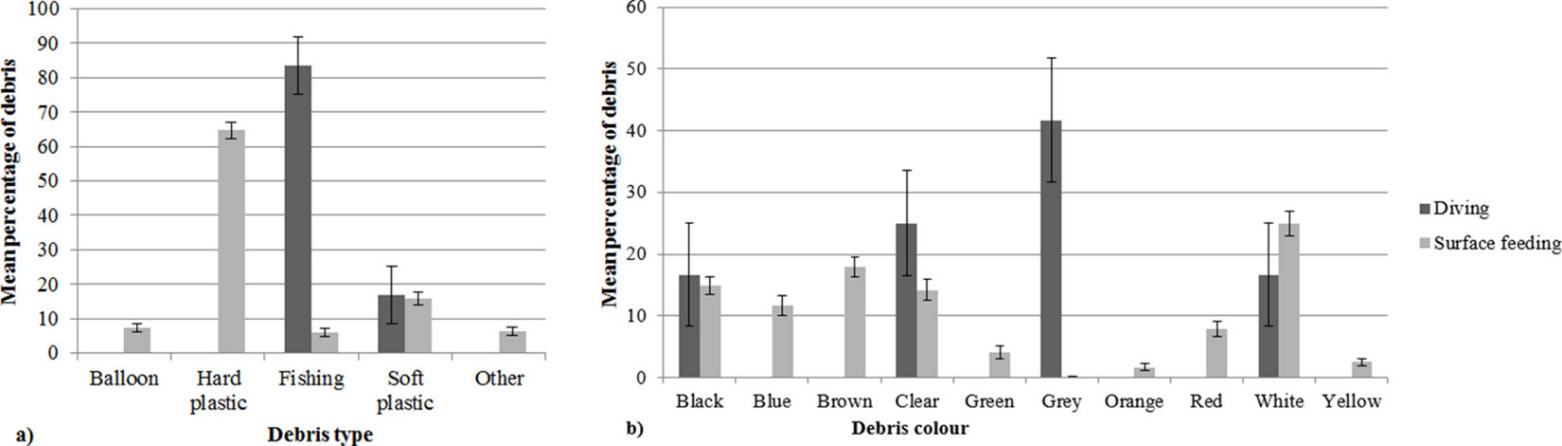
The mean percentage of the (a) type of debris (+SE) and (b) colour of marine debris (+SE) ingested by marine and coastal birds that forage by diving and surface feeding. Percentage of marine debris types ingested by marine and coastal birds differs significantly (P = 0.001) between those that forage by diving and surface feeding. The percentage of marine debris colours ingested by seabirds differs significantly (P = 0.001) between diving and surface feeding birds.

The type and colour of marine debris ingested differed significantly between diving and surface feeding foraging types (F-value = 9.82, R^2^ = 0.12, p = 0.001; F-value = 3.22, R^2^ = 0.04, p = 0.001). Debris ingested by species that forage by searching by sight was excluded due to low sample sizes. Hard plastic (64.6 ± 4.7%) was the most abundant item ingested by surface feeding birds; fishing debris, including fishing line, lures, hooks and sinkers (83.3 ± 16.7%), was the most common item ingested by diving birds (Fig 5).

## Discussion

This study is the largest survey by species of avifauna debris ingestion in the southern hemisphere. Of the 19 species found to ingest debris, ingestion had not been previously recorded in 10 of these species in Australia, highlighting a greater need to examine the breadth of debris ingestion in Australian avifauna. Our study highlighted the first scientific record of debris ingestion for four avian species, and the first record of debris ingestion in the family Burhinidae. Observation of marine debris ingestion in previously unrecorded species suggest that despite growing scientific awareness of debris ingestion among avifauna, the breadth of affected species may be greater than previously realized. Four species where debris ingestion was recorded are listed as threatened: the Westland petrel, *Procellaria westlandica* (Vulnerable), Gould’s petrel, *Pterodroma leucoptera* (Vulnerable), Buller’s albatross, *Thalassarche bulleri* (Near threatened) and Shy albatross, *Thalassarche cauta*, (Near threatened) (Table 1), drawing attention to the risk that threatened species may be further compromised by exposure to marine debris in their diets.

Many factors contribute to the likelihood of anthropogenic debris ingestion, and not all debris posed an equal risk to all birds. Our results show significant differences between the types and colours of debris ingested, suggesting that birds do not ingest debris as it is randomly encountered in their environment, but rather that birds exhibit selectivity, e.g. they may favour certain debris types and colours. Considerable variability within each group analyzed in the results, as indicated by the R^2^ values, suggest that there may be other factors contributing to variability in debris observed in each sample. While further research is required to better understand the drivers of this within-group variability, the presence of variability does not diminish the scientific value in recognizing that there is significant dissimilarity between groups examined.

For the species that ingested debris, taxonomic grouping, habitat, and foraging method influenced the types and colours of debris ingested. Understanding this complex relationship between habitat, foraging behavior, taxonomy and selectivity is important in understanding and predicting which marine birds are at higher or lower risk of anthropogenic debris ingestion. In regard to the influence of taxonomy on debris ingestion, the comparatively high prevalence and number of pieces of ingested debris per bird observed within Procellariiformes may be due to unique gizzard morphology [34]. Procellariiformes have a constricted area between the proventriculus, where undigested food is stored, and the gizzard, where food is mechanically processed. The isthmus juncture may make it more difficult for indigestible materials such as plastics to be regurgitated [34].

Many taxonomic orders of seabirds are known to disgorge pellets of indigestible materials. Suliformes, some Charadriiformes and some Procellariiformes are all known to produce boluses, with reports of plastics and other anthropogenic debris contained therein [35,36]. The ability to disgorge pellets may be associated with being less choosy when making foraging decisions for some species, as indigestible items will be regurgitated. Taxonomic orders that do not regurgitate pellets and were not observed to ingest debris in this study included Anseriformes, Gruiformes, and Podicipediformes. Furthermore, pellet regurgitation may result in an underestimation of debris ingestion if birds are necropsied after their last regurgitation. While debris was not found in terns (n = 36) or skuas (n = 1) in this study, other studies have described debris ingestion in skuas, terns, and gulls [22,34,37,38], which regularly regurgitate pellets of indigestible materials, including plastic debris [35,36].

Digestion rate of various debris items between species may also influence the types of debris observed. It has been suggested that softer debris items may be digested faster than harder debris items [39], potentially leading to an apparent hard-item selectivity bias due to the greater residence time of hard items in the digestive system.

Habitat was found to influence the prevalence of debris ingestion in studied birds. While birds in all five habitat groups examined (pelagic, coastal marine, coastal, fresh water and open grasslands) were observed to ingest debris, debris ingestion was more common among birds foraging in marine environments (both pelagic and coastal marine). Similar to other studies, pelagic species predominantly ingested hard plastics [21–23], while fishing debris was the most commonly ingested item by coastal marine species. The high percentage of fishing debris in the diet of coastal marine birds may result from foraging close to the beach where recreational fishing commonly occurs. Recreational fishing in coastal environments would increase the encounter rate of coastal marine birds with fishing debris as compared to pelagic species. The coastal fishing and coastal bird fishing-debris interaction hypothesis is supported by in-patient records from the RSPCA Wacol wildlife hospital, where a majority of the coastal seabird species brought in for treatment (e.g. Australian pelicans, cormorants and gulls) have been affected by fishing gear interactions (T. Bishop, pers. com., 10 September 2013) and observations of debris and wildlife interactions in coastal regions [40].

Marine debris ingestion has been linked to foraging behavior [21,41], an observation well supported by this study. Feeding ecology is associated with the likelihood of encountering debris within a bird’s habitat. Seabirds forage by sight both aerially and under water [42]. Therefore availability, visibility, and prey resemblance of the debris all likely play an important role in selectivity and in determining the risk of marine debris ingestion to marine birds.

Debris buoyancy may also affect availability for seabirds foraging at or near the water’s surface. Buoyant debris occurs both at and under the surface of the ocean, as it is mixed vertically by wind and ocean currents in the ocean surface boundary layer [43], where it is available to surface feeding and shallow diving birds. As may be predicted, pursuit diving species in this study ingested negatively buoyant debris (e.g. fishing hooks and sinkers), whereas surface feeding seabirds ingested positively buoyant plastics and balloons.

The visibility or detectability of debris to foraging birds may be influenced by debris colour and indeed we found that birds were selective in the colour of debris they ingested. Blue debris was the second most predominant colour sampled in trawls, yet was not a common colour of debris ingested by birds. The difference in occurrence rate of blue in ingested debris compared to trawl samples may be due to the reduced contrast of blue items against the ocean background relative to other colours. We found that white was the most commonly recorded colour of ingested debris, a result paralleled in other ingestion studies [21,23]. This may reflect the abundance of oceanic white-coloured marine debris (Fig 2). The role that bird vision and debris visibility plays in selectivity requires further study.

Prey resemblance has been thought to influence the selectivity of marine creatures. For example, marine turtles may ingest clear soft plastics because they are mistaken for scyphozoan prey [24]. Similarly, it is possible that surface-feeding birds ingesting high proportions of hard plastic may mistake the hard plastics for pelagic invertebrates, zooplankton and fish eggs. Hard plastics may also be accidentally ingested when seabirds feed on squid eggs adhered to floating plastic. It is interesting to note that balloons accounted for 2.1% of all items ingested by birds while only constituting 0.6% of all debris recorded in trawls. Of the 37 balloons fragments ingested, 54% were red/pink and 32% were orange. Short-tailed shearwaters, which ingested 82% of all balloons recorded, feed extensively on Red arrow squid, *Nototodarus gouldi* [27]. Red and orange balloons may appear similar to prey to foraging shearwaters. Selection of balloons by seabirds was also observed by Acampora et al. (2014) [19]. Selectivity due to prey resemblance is also evidenced by the occurrence of fishing lures we observed in stomach samples of diving seabirds.

Birds may also secondarily ingest debris through their prey. Plastics are known to be ingested by pelagic fishes [44,45], and seabirds that feed on these fishes can therefore ingest plastic secondarily. Foraging marine birds may feed on fish that are carrying fishing hooks, or may ingest baited fishing hooks. It is possible that some debris recorded in this study may have its origins through secondary ingestion rather than selection by birds, however we are unable distinguish primary from secondary consumption.

Anthropogenic debris is ubiquitous and dynamic in the marine environment, and travels extensively along ocean currents [46,47]. The authors acknowledge that local temporal or geographical variation in the types and colour of debris available throughout the sampled bird species foraging ranges may occur, and that specimens used were biased towards birds that were in poor health. We recognize that the trawl sampling comparison cannot provide a completely accurate representation of what debris items each bird would have encountered due to the likely spatial-temporal mismatch between sampling areas and the foraging paths of birds prior to their deaths. However, the approach is the best currently available [19,24].

Our study adds evidence of the ubiquity and prevalence of anthropogenic debris ingestion by marine avifauna. We demonstrated that ingestion in Australian marine and coastal birds is widespread and that taxonomy, habitat and foraging ecology influence debris ingestion. We found that foraging birds exhibit selectivity in the types and colour of debris ingested, with surface feeding birds ingesting higher quantities of buoyant litter items and a disproportionate amount of balloons, given their abundance in the marine environment. With the continued increase of plastic production globally and the unceasing inputs of litter to our oceans, reducing marine litter will clearly require a coordinated effort. Legislation, incentives and education have all proven to be effective [48]; finding the right mix of approaches to help resolve this growing environmental issue is clearly an important area on which to focus. Ultimately, developing a global priority list of at-risk species based upon their distribution, rates of encounter and impacts from debris will help to concentrate efforts to manage and mitigate marine debris impacts most effectively.
